# What is your diagnosis?

**DOI:** 10.4274/jtgga.galenos.2019.2019.0024

**Published:** 2019-08-28

**Authors:** Kavita Khoiwal, Anshu Gupta, K. Rupendra, Jaya Chaturvedi, Amrita Gaurav

**Affiliations:** 1 All India Institute of Medical Sciences, Rishikesh, Uttarakhand, India

An adolescent girl aged 16 years presented to the emergency department with features of shock. She had severe pallor with feeble pulse of 120/min, blood pressure: 80/40 mm Hg, respiratory rate: 22/min, peripheral capillary oxygen saturation (SpO_2_): 98%, and urine output was almost nil. Initial resuscitation was performed. The history could not be elicited from the patient herself. Her relatives revealed that she had a 4-month history of amenorrhea along with pain in the abdomen and bleeding per vaginum for the last one day. A urine pregnancy test was positive. The parents denied any history of pill intake or surgical procedures for termination of pregnancy.

The abdominal examination was within normal limits. There was no guarding, rigidity, tenderness or any palpable mass felt. Bleeding was present on local examination. A gentle vaginal examination revealed a 6x6 cm smooth, tender, round mass in the vagina, the cervical rim and uterus could not be felt. The patient did not allow a proper examination because it was very painful. An urgent blood investigation was suggestive of hemoglobin of 6.9 gm%, total leucocyte count: 28,000/cumm with normal coagulation profile. The patient was planned for examination under anesthesia (EUA).

## Answer

**Figure 1:**
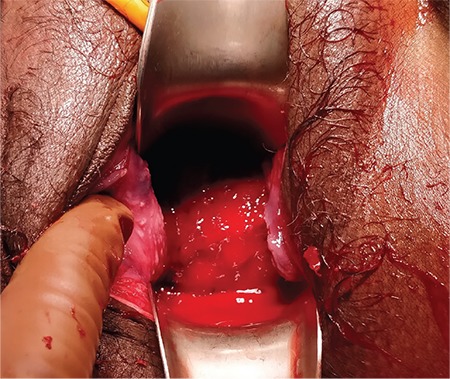
Speculum examination shows a rounded smooth mass in vagina, bleeding^++^

**Figure 2:**
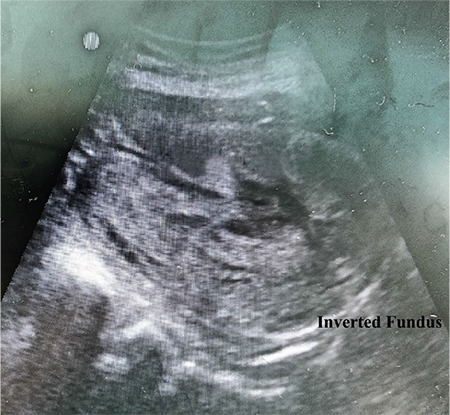
Transabdominal scan suggestive of inverted uterus

**Figure 3:**
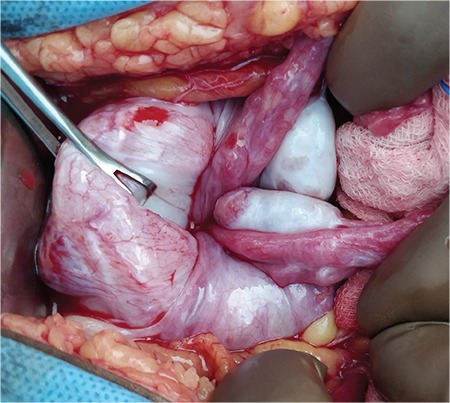
Bilateral round ligaments, fallopian tubes and ovaries seen dragged into the depression along with the upper half of the uterine body. Cervical constriction ring is seen being held with Babcock’s forceps

The patient was taken to the operation room for EUA in view of the uncertainty of diagnosis and hemodynamic instability. Informed and written consent was obtained for EUA along with emergency laparotomy if required. The remote possibility of hysterectomy was also explained. EUA was suggestive of second-degree uterine inversion, tissues were edematous and bleeding was present (Figure 1), the fundus of the uterus was not palpable on manual palpation. An intra-operative trans-abdominal scan (Figure 2) also gave rise to the suspicion of inverted uterus. Manual repositioning as well as the hydrostatic technique did not work. Laparotomy and repair of uterine inversion using the Haultain technique was performed. Intra-operatively, a cervical constriction ring with a depression was observed in place of the uterus and bilateral round ligaments, the fallopian tubes and ovaries were seen dragged into the depression along with the upper half of the uterine body (Figure 3). A vertical incision was made over the posterior aspect of the constricted cervical ring. The inversion was then corrected by following the principle ‘the part which goes first should be repositioned first’. The uterus was well retracted after correction. The incision site was repaired with delayed absorbable suture in two layers. A total of 4 units of packed red blood cells and 4 units of fresh frozen plasma was transfused to the patient. Her postoperative recovery was uneventful.

Puerperal uterine inversion is a life-threatening emergency condition that occurs after vaginal or cesarean delivery, even with hysterotomy. It has been classified on the basis of the time of occurrence from delivery (acute <24 hours, subacute 24 hours to 4 weeks, and chronic ≥4 weeks) (1). Most cases present within 24 hours of delivery (2) with severe postpartum hemorrhage followed by hypovolemic shock. In addition, neurogenic shock due to stretching of the pelvic parasympathetic nerves worsens the condition. The incidence varies in different populations, ranging from 1 in 3500 to 20,000 deliveries (3,4). There are only few case reports of uterine inversion after mid trimester abortion (5,6). Though it is a rare event, healthcare workers should be aware and vigilant about this condition because if not timely diagnosed and managed, it can lead to shock and even death.

The incidence of non-puerperal uterine inversion is further less than puerperal uterine inversion. In a systemic review of the literature (7), a total of 170 case reports of non-puerperal uterine inversion were found. The reason behind its occurrence is an polypoid tumor of uterus mostly submucosal fibroid (57.2%) followed by sarcoma (13.5%). Most of these patients (86.8%) underwent hysterectomy.

Uterine inversion is typically diagnosed through clinical findings including vaginal bleeding, lower abdomen pain, features of shock may or may not be present, inability to palpate the uterus on abdominal examination, and presence of a round smooth mass protruding from the cervix or vagina. Imaging studies are not recommended but they have a role in a few cases with uncertain diagnosis, provided that the patient is hemodynamically stable (8).

The objectives of management are to stabilize the patient by managing postpartum hemorrhage and shock, if present, and repositioning of the uterus. Prompt recognition and timely intervention is the key of management. After initial resuscitation, manual replacement of the inverted uterus should be attempted. Do not remove the placenta, if attached. If the immediate replacement maneuvers do not work, surgical methods for replacement should be considered. Surgical procedures include the Huntington procedure (giving upward traction on the inverted uterus with a clamp) or the Haultain procedure, which involves making an incision on the cervical constriction ring posteriorly to increase its size, followed by repositioning of the inverted uterus, followed by repair of the incision.

Hydrostatic reduction is an option if all other interventions have failed and surgical intervention is not possible (9).

The reported incidence of complications associated with puerperal uterine inversion are postpartum hemorrhage (38%), need of blood products (22%), laparotomy (6%), hysterectomy (3%), hypotension (2%), and shock (1.3%) (4). There are insufficient data to report the rate of recurrence in subsequent pregnancies. No recurrence was noticed in a case series (n=40) by Baskett (10).

The mode of is based upon the management option used; if the woman underwent surgical replacement with an incision over the uterus, cesarean section is a better option (11).

Puerperal uterine inversion is a rare but life-threatening condition, it may present in any woman of reproductive age. Healthcare workers should be aware and vigilant about this condition and keep it in mind whenever a woman presents with pain in the abdomen and bleeding per vaginum leading to shock in the post-partum or post-abortion period. Early diagnosis and immediate management is the key of successful outcome.
